# Malignant triton tumor of the retroperitoneum: a case report and review of the literature

**DOI:** 10.1186/1477-7819-10-96

**Published:** 2012-05-30

**Authors:** Zhiwei Li, Jie Xiang, Sheng Yan, Feng Gao, Shusen Zheng

**Affiliations:** 1 Department of Hepatobiliary and Pancreatic Surgery, First Affiliated Hospital, School of Medicine, Zhejiang University, 79 Qingchun Road, Hangzhou, 310003, People's Republic of China; 2 Key Laboratory of Combined Multi-organ Transplantation, Ministry of Public Health, First Affiliated Hospital, School of Medicine, Zhejiang University, 79 Qingchun Road, Hangzhou, 310003, People's Republic of China; 3 Key Laboratory of Organ Transplantation of Zhejiang Province, First Affiliated Hospital, School of Medicine, Zhejiang University, 79 Qingchun Road, Hangzhou, 310003, People's Republic of China

**Keywords:** Malignant triton tumor, Retroperitoneum, Surgery, Prognosis

## Abstract

**Background:**

Malignant triton tumors are relatively rare and highly aggressive tumors in which malignant schwannoma cells coexist with rhabdomyoblasts. Their occurrence in the retroperitoneum is uncommon and has been rarely reported.

**Case presentation:**

We present the case of a patient with a retroperitoneal malignant triton tumor. A 32-year-old male was referred with epigastric pain and an abdominal mass. Computed tomography revealed a huge soft tissue retroperitoneal mass that involved adjacent organs and vessels. Complete resection was undertaken. Pathological examination confirmed the presence of a malignant triton tumor. The patient died two and a half months after surgery, as a result of local recurrence.

**Conclusion:**

Malignant triton tumors are uncommon sarcomas that are associated with a high incidence of developing local recurrence and distant metastases. Immunohistochemical staining showing nerve sheath differentiation with rhabdomyoblastic cells confirms the diagnosis. Complete excision of the tumor is the most effective treatment strategy for these retroperitoneal tumors.

## Background

Malignant triton tumor (MTT) is a relatively rare subtype of malignant peripheral nerve sheath tumors (MPNST), histologically defined as a malignant peripheral nerve sheath tumor with additional rhabdomyoblastic differentiation. MTTs are usually located in the head, neck, extremities and trunk [1]. Less common sites include the buttock, the mediastinum and the retroperitoneum [2]. Tumors in the retroperitoneum, however, are extremely rare, and fewer than 10 cases have been reported since 1984 [1,3-8]. We present the case of a retroperitoneal MTT in a 32-year-old male patient, and review all published clinical reports of this tumor to date.

## Case presentation

A 32-year-old male, previously fit and healthy, presented with epigastric pain and was found to have an abdominal mass on abdominal ultrasound. He was referred to the First Affiliated Hospital, School of Medicine, Zhejiang University for further investigation and treatment in October 2010. There were no signs of neurofibromatosis type 1(NF-1). Physical examination revealed a firm, ill-defined, fixed mass in the upper abdomen. Laboratory findings, including leukocyte and platelet counts, hemoglobin, serum creatinine, liver function, alpha-fetoprotein, carcinoembryonic antigen and cancer antigen 19–9, were all within normal limits.

**Figure 1:**
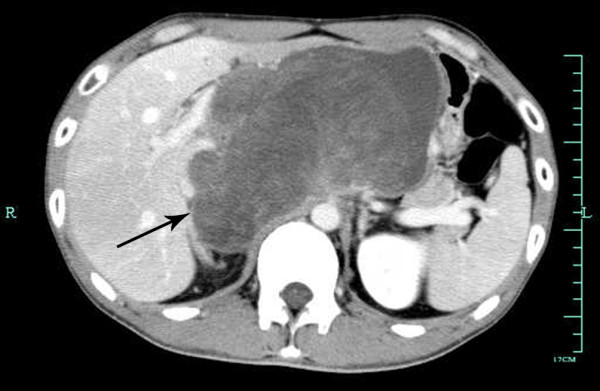
Abdominal computed tomography showing a huge tumor occupying the entire retroperitoneal space.

Contrast-enhanced computed tomography (CT) of the chest and abdomen showed a heterogeneous tissue retroperitoneal mass, approximately 16 cm in diameter (Figure 1). Following contrast, heterogeneous enhancement of the mass was noted. Adjacent vessels, such as the common hepatic artery, the portal vein and the inferior vena cava, were compressed. There was no evidence of associated lymphadenopathy or distant metastases. Biopsy of the tumor suggested a soft tissue sarcoma composed of pleomorphic spindle cells.

All imaging studies and serology examinations indicated that surgery was feasible. At surgery, a huge, soft, whitish, solid and cystic tumor was found, which occupied the entire abdomen. The tumor appeared to involve the distal stomach, the diaphragm, the hepatoduodenal ligament, the gastrohepatic ligament, the left lobe of the liver and the celiac trunk. It was also compressing the walls of the abdominal aorta and the inferior vena cava. Massive varicose veins were noted in the abdominal cavity. Part of the left lobe of the liver was resected due to tumor infiltration. A distal gastrectomy with a Billroth II anastomosis was simultaneously performed due to tumor involvement of the stomach. In addition, we suspected that the common bile duct had also been invaded by tumor, which was not evident on preoperative imaging, and we therefore performed a resection of the common bile duct, a cholecystectomy and T-tube drainage. Intraoperative histological examination of a frozen section suggested the presence of a soft tissue sarcoma.

On gross examination, the tumor measured 16 × 15.5 × 8.2 cm. The cut surface appeared firm and yellowish in the peripheral portion with foci of hemorrhage and excessive necrosis in the center. The margins appeared macroscopically clear.

**Figure 2:**
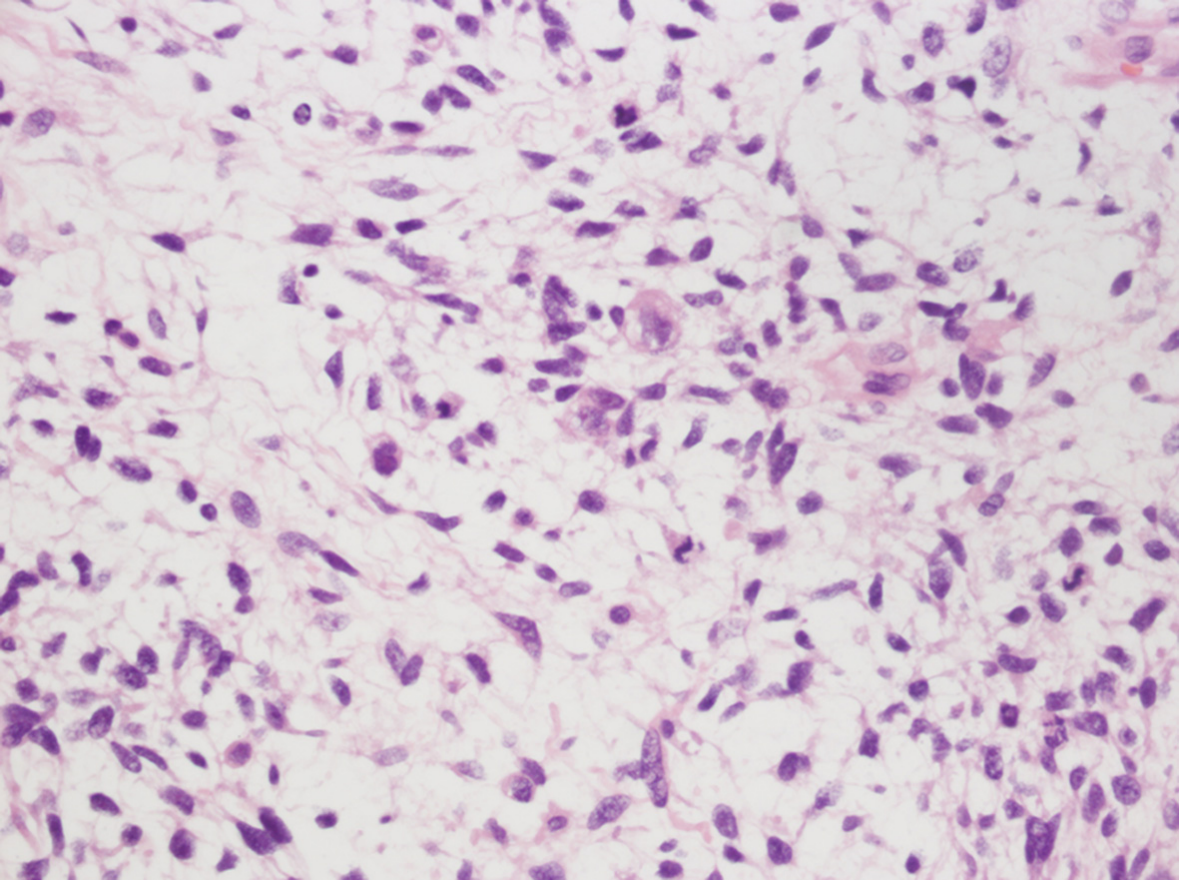
**Images of HE staining showing typical malignant schwannoma cells and rhabdomyoblasts.** Large pleomorphic rhabdomyoblastic cells with abundant eosinophilic cytoplasm are embedded in a spindle cell tumor with a fine fibrillary matrix. (Original magnification 400×).

On further histopathological examination, the neoplastic tissue displayed interlacing fascicles of spindle cells with wavy, elongated hyperchromatic nuclei. There was pronounced pleomorphism, an increased mitotic index (>50 mitoses per 10 high-power fields) and hypercellularity. Rhabdomyosarcomatous differentiation was evidenced by foci of scattered, round cells with a prominent eosinophilic cytoplasm and atypical nuclei, which were identified as rhabdomyoblasts (Figure 2). Microscopically, the margins were confirmed to be clear of residual tumor.

**Figure 3:**
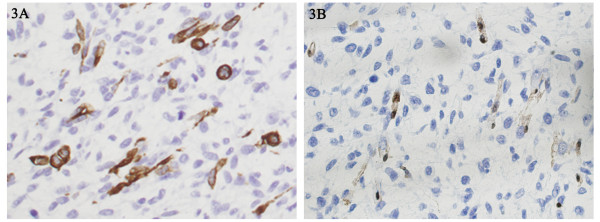
**Immunohistochemical features of desmin and S-100 staining in the malignant triton tumor (A and B).** The mass was positive for desmin (A) and S-100 (B). (Original magnification 400×).

Immunohistochemistry demonstrated positive staining of the rhabdomyoblastic cells for desmin (Figure 3A) and vimentin. Nerve sheath differentiation of the spindle cells was confirmed by S-100 protein (Figure 3B) positivity. Tumor tissue was negative for smooth muscle actin, HMB-45, CD34 and CD117. Based on these findings, the diagnosis of a MTT was confirmed.

According to the Fédération Nationale des Centres de Lutte Contre le Cancer (FNCLCC) grading system [9], the tumor was classified as grade 3 (a total score of 7: 3 for malignant triton tumor, 3 for >50 mitoses per 10 high-power fields and 1 for <50% tumor necrosis). The surgical margins were estimated as wide [10]. As no macroscopic or microscopic tumor remained, surgical resection was judged complete.

Following surgery, the patient made a good recovery and was discharged two weeks later. However, during his first follow-up assessment one month after surgery, CT imaging revealed multiple lesions in the liver, which were considered a recurrence of the tumor. Clinical deterioration occurred rapidly, and no subsequent treatment was undertaken. The patient died two and a half months after the operation.

## Discussion

Rhabdomyosarcomatous elements within MPNSTs were first described by Masson in patients with neurofibromatosis [11]. The name triton was first used by Woodruff *et al.*[12] on the basis of the discovery that supernumerary limbs containing neural and muscular elements were induced to grow on the backs of triton salamanders by transplantation. Woodruff *et al.*[12] also proposed three criteria for determining whether a neoplasm could be truly classified as a malignant ‘Triton’ tumor or not: one, arises from a peripheral nerve, or in a patient with NF-1, or in a location typical for peripheral nerve tumors, or represents a metastasis from such tumor; two, demonstrates the growth characteristics of Schwann cells; and three. contains bona fide rhabdomyoblasts that appear to arise from within the body of the peripheral nerve tumor and which cannot be attributed to either an extension or metastasis from an extrinsic rhabdomyosarcoma. Daimaru *et al.*[13] later suggested broadening the definition to include the following: one, tumors in patients without NF-1 that are microscopically compatible with a malignant schwannoma and contain focal rhabdomyoblasts; and two, tumors consisting predominantly of rhabdomyoblastic differentiation with focal Schwann cell elements occurring within a nerve or in patients with NF-1. Today, the diagnosis of MTT is generally made according to these criteria, as well as on immunohistochemistry findings [14]. The presence of positive immunoreactivity to S-100 protein or Leu-7 indicates nerve sheath differentiation, while rhabdomyoblastic cells have a positive immunoreactivity to desmin, muscle specific actin, myosin, vimentin, and myoglobulin [2,12,14]. In our case, we identified typical malignant schwannoma cells and rhabdomyoblasts. Furthermore, immunohistochemistry was positive for desmin, vimentin and S-100 protein, confirming the diagnosis of MTT.

Epidemiologically, the mean age of patients with MTT has been estimated to be 31.7 years [2,4], although the disease may affect anyone between the ages of 0 and 81 years. There appears to be an equal sex distribution in those affected, and the tumor coexists with NF-1 in 44% to 69% of cases. When associated with NF-1, MTT tends to present at a younger age and in males, than has been observed in sporadic cases. MTT is a highly aggressive tumor with low overall survival rates (estimated five-year survival rates are 26%) [2] and high rates of metastases (48%) and local recurrence (43%) [1,12]. The main factors affecting survival appear to be the location of the tumor and the extent of the excision [1,2,12,13,15]. Patients with head, neck or extremity neoplasms survive longer than those with tumors of the buttock, trunk or retroperitoneum [2]. Complete resection appears to be associated with an improved prognosis, decreased rates of local recurrence and metastasis, and a better response to adjuvant therapies; however, most patients with MTTs die even after receiving all available treatments, usually within months [1,2]. The present case was a 32-year-old male patient without a history of NF-1. The tumor was huge (16 cm in diameter) and located in the retroperitoneum, with involvement of adjacent organs. Despite complete resection, the tumor progressed and recurred within a month.

To date, approximately 170 cases of MTT have been reported in the literature worldwide [2,16,17]. Among these, MTT located in the retroperitoneum is extremely rare. We found only eight cases reported in the English literature using MEDLINE. Including the present case, data from nine patients have been reviewed. The clinical characteristics of these cases are summarized in Table 1. All patients presented with a huge retroperitoneal mass, which ranged from 7 to 19 cm in diameter. The size at presentation may be explained by the fact that retroperitoneal tumors are often asymptomatic in the early stages, as demonstrated in our case. The presence of a retroperitoneal MTT may only be expressed by the insidious onset of nonspecific and late symptoms, such as vague abdominal pain due to compression or infiltration of adjacent viscera, vessels, or nerves. Thus retroperitoneal MTTs are associated with a worse prognosis, higher rates of metastatic disease and earlier recurrence rates, than MTTs in the extremities, due to delays in diagnosis, similar to MPNSTs in the abdominal cavity [7,18]. The patient we present had a long history of postprandial abdominal distention and dull pain. Symptoms such as abdominal pain followed when the tumor had grown, measuring 16 cm in diameter, at which point it was associated with infiltration of adjacent viscera and vessels.

**Table 1:** Summary of malignant triton tumor cases of the retroperitoneum

**Case**	**Sex/Age**	**PastHistory**	**Treatment**			**Tumor Size**	**Metastases/Recurrence**	**Follow up**
			**Surgical Resection**	**Chemotherapy**	**Radiotherapy**			
Ducatman1984 [3]	12/F	NF-1	Complete	-	-	N/A	Recurrence 2 mo after surgery	DOD 15 mo
Brooks1985 [1]	Congenital/F	N/A	Incomplete	VCR,ACT,CTX	0.9 Gy	9 cm	Liver, pleura metastases	DOD 3 yr
	33/M	NF-1	Incomplete	ADM		‘large’	Lung metastases	DOD 14 mo
Haddadin 2003 [4]	81/M	Paget’s disease of bone	N/A	N/A	N/A	15 cm	Small and large bowel, peritoneum metastases	N/A
Rekhi 2008 [8]	23/F	-	Incomplete	+(unspecified)		16 cm	No metastasis	N/A
Radovanovic,2008 [7]	60/F	-	Complete	-	-	7 cm	Rectum, lungs, liver, spleen metastases, local recurrence 1 mo after surgery	DOD 2 mo
Hoshimoto2009 [5]	21/M	NF-1	Complete	+(unspecified)		19 cm	Psoas muscle, thigh, hip joint, abdominal aorta, lung metastases	DOD 14 mo
Koutsopoulos2011 [6]	18 F	NF-1	Complete	IFO,ADM	54 Gy	9.5 cm	No metastasis/recurrence	ANED >5 yr
Present case	32/M	-	Complete	-	-	16 cm	Liver, stomach, abdominal aorta metastases	DOD 2.5 mo

ACT, dactinomycin; ADM, Adriamycin; ANED, alive, no evidence of disease; CTX, Cytoxan; DOD, dead of disease; IFO, Ifosfamide; VCR, vincristine.

Due to the rarity of MTT, treatment modalities, especially in advanced and metastatic cases, are not well established, and there are no standardized management guidelines. As with many soft tissue sarcomas (STSs), the most effective therapeutic strategy for retroperitoneal MTT is complete resection of the tumor. The status of the microscopic margins after resection has a significant impact on local outcome and survival [10]. Enneking *et al.*[10] described four types of margins histologically: intralesional, marginal, wide and radical. However, surgical margins are often difficult to define as metastasis within the abdomen may occur. Furthermore, the retroperitoneal space is one of the most challenging sites technically. Anatomic constraints limit the ability to achieve wide excision in this location, which is unlikely to be attained in most cases. This creates the challenge of balancing the need for adequate margins for optimal tumor control with the need to minimize operative morbidity and loss of function, similar to other STSs [19]. In our patient, the primary tumor was huge and involved adjacent organs. We performed complete resection with wide margins, at the cost of the excision of invaded organs, including the liver, stomach, gallbladder and common bile duct. However, local recurrence still developed soon after surgery before postoperative treatment could be commenced.

The role of adjuvant therapy, for example radiotherapy and chemotherapy, is less well defined and has not been proven to be effective [1,14,15], although it may be of value in individual patients who have undergone complete resection [6,20-22]. Several reports suggest that repeated resection combined with chemotherapy and/or radiotherapy for recurrent MTTs may prolong survival [6,20,21]. The only patient with a retroperitoneal MTT who experienced long-term survival (>5 years) in our review was reported to have undergone a complete resection followed by postoperative radiotherapy and chemotherapy. Moreover, neoadjuvant chemotherapy in MTT has been reported in two patients with metastatic disease [23], although the number of cases is too small to allow a definitive conclusion on the effectiveness of neoadjuvant chemotherapy.

## Conclusion

Malignant triton tumors are uncommon sarcomas that are associated with a high incidence of local recurrence and distant metastases. Immunohistochemical staining showing nerve sheath differentiation with rhabdomyoblastic cells confirms the diagnosis of MTT. Complete excision of the tumor is the most effective therapeutic strategy for retroperitoneal MTTs.

## Consent

Written informed consent was obtained from the patient for publication of this case report and the accompanying images. Copies of the written consent form are available for review upon request.

## Abbreviations

CT, computed tomography; FNCLCC, Fédération Nationale des Centres de Lutte Contre le Cancer; MPNST, malignant peripheral nerve sheath tumor; MTT, malignant triton tumor; NF-1, neurofibromatosis type 1; STS, soft tissue sarcomas.

## Competing interests

The authors declare that they have no competing interests.

## Authors’ contributions

LZW formulated the manuscript, XJ prepared the histological figures, YS and GF provided the clinical history and clinical figures, ZSS is the guarantor. All authors read and approved the final manuscript.

## Funding

The work is supported in part by the Foundation for Innovative Research Groups of the National Natural Science Foundation of China (No.81121002) and the Zhejiang Provincial Natural Science Foundation (No.J20100398).
